# Plant-Based Phytochemicals as Possible Alternative to Antibiotics in Combating Bacterial Drug Resistance

**DOI:** 10.3390/antibiotics9080480

**Published:** 2020-08-04

**Authors:** Hana Mohammed Al AlSheikh, Insha Sultan, Vijay Kumar, Irfan A. Rather, Hashem Al-Sheikh, Arif Tasleem Jan, Qazi Mohd Rizwanul Haq

**Affiliations:** 1 Department of Prosthetic Dental Sciences, College of Dentistry, Kind Saud University, Riyadh P.O. BOX 145111, Saudi Arabia; halashaikh@ksu.edu.sa; 2 Department of Biosciences, Jamia Millia Islamia, New Delhi 110025, India; inshasultan12@gmail.com; 3 Department of Biotechnology, Yeungnam University, Gyeongsan, Gyeongbuk 38541, Korea; vijaykumarcbt@ynu.ac.kr; 4 Department of Biological Sciences, Faculty of Science, King Abdul Aziz University, Jeddah P.O. BOX 80200, Saudi Arabia; ammm@kau.edu.sa; 5 Department of Biological Sciences, College of Science, King Faisal University, Al-Ahsa 31982, Saudi Arabia; plxha@kfu.edu.sa; 6 School of Biosciences and Biotechnology, Baba Ghulam Shah Badshah University, Rajouri 185234, India

**Keywords:** antibiotics, bacteria, human health, plant-derived substances, resistance

## Abstract

The unprecedented use of antibiotics that led to development of resistance affect human health worldwide. Prescription of antibiotics imprudently and irrationally in different diseases progressed with the acquisition and as such development of antibiotic resistant microbes that led to the resurgence of pathogenic strains harboring enhanced armors against existing therapeutics. Compromised the treatment regime of a broad range of antibiotics, rise in resistance has threatened human health and increased the treatment cost of diseases. Diverse on metabolic, genetic and physiological fronts, rapid progression of resistant microbes and the lack of a strategic management plan have led researchers to consider plant-derived substances (PDS) as alternative or in complementing antibiotics against the diseases. Considering the quantitative characteristics of plant constituents that attribute health beneficial effects, analytical procedures for their isolation, characterization and phytochemical testing for elucidating ethnopharmacological effects has being worked out for employment in the treatment of different diseases. With an immense potential to combat bacterial infections, PDSs such as polyphenols, alkaloids and tannins, present a great potential for use, either as antimicrobials or as antibiotic resistance modifiers. The present study focuses on the mechanisms by which PDSs help overcome the surge in resistance, approaches for screening different phytochemicals, methods employed in the identification of bioactive components and their testing and strategies that could be adopted for counteracting the lethal consequences of multidrug resistance.

## 1. Introduction

Antibiotics are natural or synthetic organic molecules that are effective against microorganisms. With propensity to human use as defense armor, they provide protection against infections caused by bacteria and fungi. The emergence of resistance among microbes has limited the effectiveness of antibiotics, shifting the lifesaving paradigm built around them. This prominent trend is regarded as a matter of concern by agencies like the World Health Organization and has become a challenging situation for the medical fraternity worldwide [1]. Despite incredible advancement in the scientific and modern medicine, the hopefulness of antibiotics is faded away by the appearance of different resistance mechanisms that presents a grave concern against the frontline antibiotics. Of them, antibiotic inactivation via, production of a range of enzymes, change in cell permeability, alteration of drug targets, intrinsic expression of efflux pumps and biofilm formation that acts as defense against drugs and contribute to sustained persistence of resistance bacteria [2,3,4,5,6,7]. In addition to mutations, mobile genetic elements (plasmids, insertion sequences, transposons and integrative conjugative elements) play a crucial role in the expansion of resistance among diverse groups of bacteria [8]. Occurrence of resistance genes in bacteria favors their propagation and perpetuation in new territories. Contributing a unique niche for the growth of microorganisms, their establishment in the oral cavity especially dental surfaces serves as etiological agents of dental caries [9,10,11]. There were a large number of reports that suggest irrational enhancement in prescribing antibiotics by medical professionals including dental surgeons that in turn contributes significantly amount to the growing burden of microbial resistance [12,13,14]. Confronted by increasing amounts of antibiotics use, appearance of opportunistic organisms intrinsically resistant to drugs that thwarts currently available treatment regimes makes diseases difficult and, sometimes, impossible to treat in hospitals and the community settings.

An extensive increase in antibiotic resistance owed to a sustained persistence of resistant bacteria is becoming a serious threat for public health worldwide. The situation is aggravated by the decline in the production of drugs since the late 1960 s and by the long periods of time needed for testing new drugs before acceptance by the authorities for commercialization [15]. Under these circumstances, there is a growing need for identifying alternatives to antibiotics in the prevention and treatment of microbial infections. One such approach is the utilization of naturally occurring botanicals that have the potential to be used as an alternative or a complement to antibiotics. Conventional curative systems have counted upon traditional herbs that are rich in compounds, such as alkaloids, terpenoids, tannins, steroids, coumarins and flavonoids [16], and do not normally cause resistance [17]. Essential oils (Eos) from parsley, lovage, basil and thyme disrupt the physiological status of the bacterial cell by causing an increase in cell permeability, leakage of cell constituents, alterations in bacterial cell wall and cell membrane, ATP loss, inhibition of protein synthesis, pH disturbance, intracytoplasmic damage, DNA damage and inhibition of quorum sensing among bacteria like *Bacillus cereus*, *Staphylococcus aureus*, *Pseudomonas aeruginosa*, *Escherichia. coli* and *Salmonella enterica* serovar Typhimurium [18,19]. The present review summarizes the approaches for screening different phytochemicals, methods employed in the identification of bioactive components and their testing, strategies adopted along with the mechanistic insights of plant-derived substances (PDSs) in counteracting the lethal consequences of multidrug resistance.

## 2. Isolation and Characterization of Bioactive Compounds

Different approaches are employed for screening the bioactive constituents of PDSs. The direct method involves total chemical characterization of a plant species using approaches of dereplication (identification of already known bioactive constituents) via liquid chromatography–mass spectrometry (LC–MS). The objective is to isolate novel plant compounds, which are then sent directly for biologic testing [20,21,22,23,24]. Isolated plant compounds are collected for libraries, such as the National Cancer Institute’s Natural Product Repository [25]. Libraries can be screened to test the bioactivities of various compounds in order to examine fully characterized, new natural products [26,27,28]. Bioactivity-guided fractionation method uses bioassays as fractionation monitors; uninterrupted fractionation cycles are used together for testing bioactive extracts so that pure and active principle compounds are isolated. This is the most commonly used way of determining bioactives. Despite significant increase in the number of studies reporting bioactives [29,30,31,32] through this process, a large number of botanicals and their preparations are still in use without any knowledge of their bioactives [33,34,35,36]. The synergy directed fractionation method uses bioactivity-guided fractionation combined with bioactivity testing in order to understand synergistic connections between the compounds present in a mixture. The method uses mass spectrometry (MS)-profiling for a guided isolation of natural products, targeting the synergistic interactions among obtained extracts which could not have been taken into consideration through conventional guided fractionation [37,38,39,40,41]. Metabolism directed method is focused on the identification of bioactive metabolites not present at the beginning of the test, but produced with time due to changes in the metabolism of the plant or plant organ under study [42,43,44,45,46,47,48,49]. Additionally, metabolic profiling (metabolomics) method correlates chemical profiling of plant extracts with isolation and identification of new or already known bioactive constituents at an early stage (dereplication) [50,51]; the goal of metabolomics in general is to analyze all secondary metabolites in a sample, both qualitatively and quantitatively. Comparison of bioactivity data permits early stage dereplication. This method also gives information about possible synergistic effects between molecules [52,53,54,55,56,57,58].

## 3. Screening of PDSs for Drug Discovery

Plant materials are selected on the basis of habitual interactions between plants and the environment, taking into consideration that synthesized secondary metabolites may possess significant therapeutic benefits for humans. The screening of PDSs for drug discovery includes random, ethnopharmacological and computational approaches. Random selection of extracts is based on accessibility of plants and their enriched fractions. It unravels unexpected bioactivities that could have been missed based on the present knowledge [59,60,61,62,63]. As pharmacological assays have medium to small throughput and the starting test samples (extracts, fractions or pure compounds) are available only in small amounts, the number of bioassays through which they can be tested is limited. The ethnopharmacological approach relies mainly on the traditional medicinal applications of plant species [34,64,65,66,67]. This approach uses the observation, description and experimental investigation of traditionally used drugs and their bioactivities. It involves an interdisciplinary approach, including botany, biochemistry, chemistry, pharmacology and other fields beyond natural sciences like anthropology, archeology, history and linguistics [68,69,70]. In computational approach, plant materials or natural products are selected based on the prediction of their bioactive constituents with a high likelihood for biologic activity. In addition, to predict characteristics of molecular structures like protein–ligand biding interactions, in silico simulations can be used. Various computational approaches have been reported, such as the QSAR model (quantitative structure activity relationship); [71,72], which is used to predict compounds with high specificity for targets. Bioactivity databases, such as CHEMBL [73] or PubChem [74] are used to evaluate datasets for compatibility among naturally obtained products and synthetically obtained pharmacologically active molecules. The pharmacophore model [75,76] simulates 3D arrangements of molecules with different physicochemical features involved in the interactions between a ligand and its target. Meanwhile, molecular docking has a crucial function in defining drug–protein interactions, helpful for determining whether plant extracts/compounds act as substrates for efflux pump proteins. Molecular docking suggests structure–activity relationships of natural products for elucidating their mechanism of action and predicting the positioning of a ligand within a protein-binding pocket [77].

## 4. Bioassays for Phytochemical Testing

Gas chromatography–mass spectrometry (GC–MS) techniques are commonly used for the analysis of phytochemicals in plant extracts [78,79,80,81,82]. Due to its high selectivity and sensitivity in the analysis of chemical constituents of plant origin, GC–MS is considered as the gold standard for the elucidation of phytochemical profiles of plant extracts. In addition, agar macrodilution and microdilution methods are generally used to determine the minimum inhibitory concentration (MIC) of plant extracts against different bacterial isolates (Table 1).

Though screening of phytochemicals for antimicrobial activity is usually done by the agar well diffusion method, time-kill assay and lysis experience, commonly employed bioassay methods and new methodologies adopted in drug discovery includes in vitro assays (based on purified proteins, cell and target oriented), in situ assay (assay with isolated tissues and organs) and in vivo assays with model organisms.

### 4.1. In Vitro Assays 

**Figure 1:**
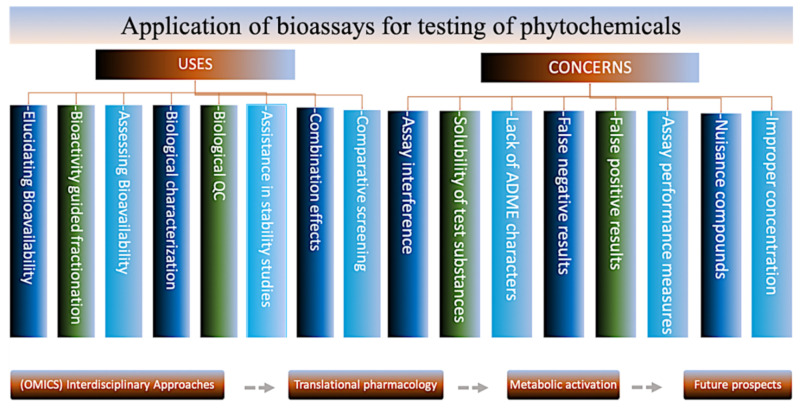
Bioassays for testing Phytochemicals—Uses and concerns.

Assay with purified proteins relies on measuring either the pattern of interaction of a test compound with the target protein or the functional activity of the target protein in the presence of a test compound [64] (Figure 1). Allowing high throughput screening, it does not require cell culture or animal facilities. The method has a limitation of showing unrelated interaction between protein and the compound being tested. Cell-based assay is used to verify the activity of compounds at the cellular level. It may also unveil molecular mechanisms underlying certain biologic effects, leading to new target molecule discovery or pathways affecting the respective phenotype [64]. It provides medium to high throughput data and shows hits at the cellular level that are helpful in understanding the mechanisms of action involved and that can be used to discover novel molecular pathways to study concerned phenotypes. However, the method demands maintenance of the cell cultures and requires thorough knowledge and tedious efforts to reveal molecular targets affected in order to learn about the changed phenotypes and does not provide efficiency at in vivo level. Cell-based target oriented assay provokes the inhibition of a protein–protein interaction to check the functional activity or the activation of a protein upon binding to a compound [64]. The method provides knowledge of the molecular targets that helps in understanding the mechanism of action. It allows high throughput screening, demonstrating the efficiency of a target and giving information at a cellular level. The method has a prerequisite of cell culture and does not guarantee in vivo effectiveness of the compound being tested.

### 4.2. In Situ Assay with Isolated Tissues or Organs

The assay works at the interface of in vitro and in vivo models, encompassing an in situ method for isolated tissues or organs [103,104]. The method holds a high pathologic and physiological significance that reduces animal usage and allows high throughput in contrast to rodent models. However, the method provides low throughput in contrast to cell-based assays and have issues related to ethical clearance due to animal usage. Additionally, the isolated tissues and organs have a short ex vivo half-life.

### 4.3. In Vivo Assays Based on Model Organisms

Rodent models are employed for checking the in vivo efficacy of a drug, the discovery of new pharmacological targets and in the elucidation of the mechanisms of action of pharmacologically active compounds [105,106,107]. Having high homology with respect to humans, the method promises of the high pathophysiological significance of hits in a whole organism. Limitations of the method include slow throughput, requirement of ethical clearance and demand of having animal facility. Much time is needed to follow-up the work until the identification of the targets at a molecular level is achieved. *Caenorhabditis elegans* and the zebrafish (non-mammalian animal models) are next in the usage due to their widespread availability and the existence of instruments that support high throughput screening [108,109]. Their applicability as disease models is further expanded by the recent implementation of gene editing techniques [110]. These models are of high pathophysiological importance as they involve whole organisms. They could give rise to transgenic models. They require less amounts of the test substances in comparison to rodent models and are cost-effective. However, much time is needed to follow up on the work until the identification of the affected molecular target, besides detecting species-specific effects that are not relevant to humans. *Galleria mellonella* is used for the evaluation of pathogenesis and is used to study the potential of antimicrobial compounds. *Galleria mellonella* provides advantages of being used to test various bacterial species in a low time range with the cost-effectiveness manner. The management of this model comes easy. These qualities make *Galleria mellonella* an excellent model to check the in vivo efficacy of novel therapeutic remedies and simultaneously studying the host–pathogen interactions. *Galleria mellonella* is still at its infancy because the procedures for this model are not fully standardized [111].

## 5. Mechanistic Insights of Research on Botanicals

Plants synthesize a huge collection of structurally different compounds (polyphenols, terpenoids, essential oils, lectins, polypeptides and alkaloids), each having a specific and distinct role in the defense of the plant. Serving as potent contenders for future medicines, plant-based metabolites interfere with bacteria through different mechanisms. These are listed below.

### 5.1. Inhibition of Cell Wall Synthesis

Peptidoglycan is composed of repeating units of *N*-acetylmuramic acid (NAcMur) and *N*-acetylglucosamine (NAcGlc) residues cross-linked by short chains of amino acids. The sequence of amino acid residues plays a crucial role in terms of providing strength, and as such, protection to bacteria [112]. Plant-derived compounds have proved useful in the improvement of therapeutic methods to control the synthesis of the bacterial cell wall. The stamped antimicrobial property of flavonoids against an assortment of bacterial and contagious pathogens is attributed towards their action on the microbial cell wall [113]. Their interaction with membrane proteins attached with bacterial cell walls prompts expanded membrane penetrability and disturbance. Quinone, for example—anthraquinone from *Cassia italica*—was observed to be bacteriostatic against pathogenic microbes like *Bacillus anthracis*, *Corynebacterium pseudodiphthericum* and *Pseudomonas aeruginosa* and bactericidal towards *Burkholderia pseudomallei*. Cell wall lysis has likewise been recorded in bacteria presented to phenolic mixes. Flavones, flavonoids and flavonols, their action is most likely because of their capacity to complex with bacterial cell walls along with the extracellular and dissolvable proteins (Table 2).

**Figure 2:**
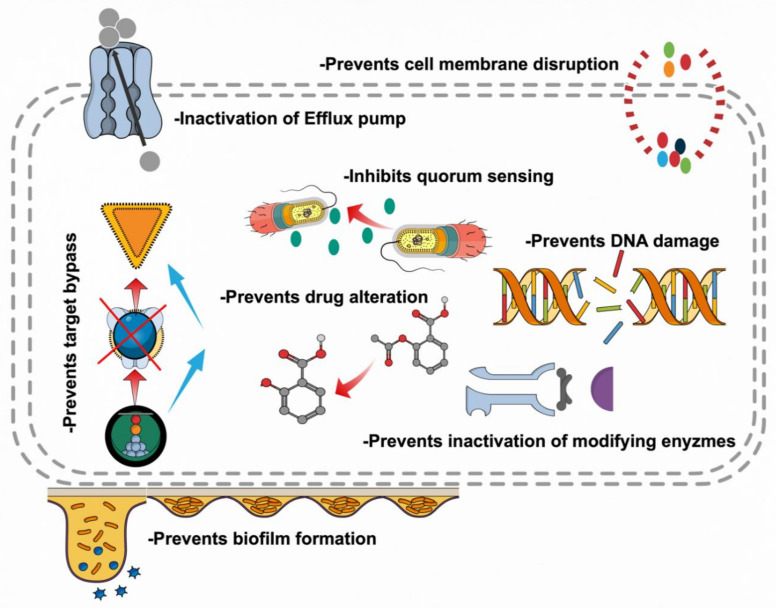
Phytochemicals as antimicrobials—mode of action and their effectiveness against microbes.

Increasing lipophilic flavonoids may likewise upset bacterial membranes. Investigation with scanning electron microscopy (SEM), subsequent to applying the flavonoid glycosides, showed that *Pseudomonas aeruginosa* cells began to distort at 30 min. At 60 min, the cells were totally twisted, in this manner recommending that the system of activity is through framing pores in the cell wall and harming the cell wall [126]. Tannins have properties that repress the development and protease action of ruminal microorganisms by targeting cell wall of bacteria. They could likewise disturb cell layers if lipophilic enough [127]. Alkaloids for the most part display antimicrobial action through intercalating into the cell wall and DNA of bacteria. At the point when galangin was joined with amoxicillin, transmission electron microscopy uncovered the detachment of the external membrane of the cells; a conceivable instrument is harm to the inside peptidoglycan layer. Amoxicillin and kaempferide or kaempferide-3-O–B-D-glucoside had an expanded hole between the external and cytoplasmic layers. These cells additionally exhibited morphologic harm to the cell wall and its shape. A few bacteria had broken cell walls. Amoxicillin or flavonoids alone were unfit to adjust the penetrability of the external layer as opposed to their blends. The impacts of consolidating amoxicillin and kaempferide or kempferide-3-O–B-D-glucoside were more powerful than amoxicillin and galangin [128,129]. Increment in porousness of membrane and disturbance is seen because of their association with bacterial cell wall as well as with membrane proteins present on it (Figure 2).

### 5.2. Inhibition of Bacterial Physiology

Though the mechanisms of action of plant-derived substances (PDSs) is not clear, it is believed that they interfere with the organization of the cellular membrane leading to a diminished membrane potential and lower levels of ATP synthesis. Addition of PDSs to the medium causes changes in membrane permeability, metal ion chelation and disruption in the activity of membrane-bound ATPase that changes the physiological state of the bacteria and resulting in bacterial death [130,131,132,133,134,135]. It has been observed that thymol, carvacrol, catechins and eugenol disrupt the membrane structure and cause the discharge of cellular components, leading to depletion of cellular ATP [132,133,134,135]. Cinnamaldehyde also provokes a depletion of intracellular ATP by hampering ATPase-dependent metabolism, besides inhibiting glucose uptake and consumption [132,133,134,135]. Moreover, tea tree oil, composed of terpenes, monoterpenes, sesquiterpenes and the alcohols 1,8-cineol, α-terpineol and terpinen-4-ol, has the capacity to disrupt membrane permeability, damage cell membrane and obstruct cell growth, causing cell death in resistant microbes, like *Escherichia coli*, *Staphylococcus aureus* and *Candida albicans* [136].

### 5.3. Modulation of Antibiotic Susceptibility

Researchers has discovered that PDSs have a built-in antimicrobial potency and can be effective as resistance modulating compounds. Geraniol, an essential oil from *Helichrysum italicum*, can reinstate the efficiency of chloramphenicol, quinolones and β-lactams towards multidrug resistant bacteria *Acinetobacter baumannii* [137]. Comparable synergism was seen among antibiotics and several medicinal plant extracts, such as those from *Camellia sinensis* [138] and *Cesalpinia spinosa* [139], the oil of *Croton zehntneri* [140] and compounds like carvacrol [141] and baicalein derived from *Scutellaria baicalensis* [142].

The control mechanisms of plant compounds for bacteria are being studied due to their participation in reducing the effect of strategies used by bacteria to combat the effects of antibiotics (including enzymatic degradation and alteration of target sites and efflux pumps (EPs) [143]. Moreover, the combination of antibiotics and PDSs may be used in therapies to inhibit many pathways required for normal bacterial infection mechanisms and for reducing the synthesis of bacterial virulence factors. While extracts from *Bridelia micrantha* and *Garcinia lucida* restrict activity of β-lactamases [144], inhibition of the penicillinase activity by epigallocatechin gallate from green tea in methicillin-resistant *Staphylococcus aureus* (MRSA) restores antibacterial properties of both penicillin and ampicillin [145]. As several PDSs were found having an inhibitory effect on EPs, investigations on eugenol, β-resorcylic acid, trans-cinnamaldehyde, thymol and carvacrol (individually, as well as in different combination) showed that they increased the sensitivity of *Salmonella enterica* serovar *Typhimurium* DT104 towards five antibiotics [146].

Although the essential oils (EOs) of *Chrysanthemum coronarium* L. have no activity against *Escherichia coli*, *Staphylococcus aureus* or *Pseudomonas aeruginosa*, its application together with ticarcillin, imipenem, gentamicin and tobramycin in culture media results in a decrease in the antimicrobial activity of these antibiotics against most bacteria tested;, but a synergistic effect was also detected against some other bacteria [147,148]. Furthermore, as antimicrobial resistance in *Salmonella enterica* serovar *Typhimurium* DT104 is attributed to the interaction of antibiotic transporters with the efflux system AcrAB–TolC, it becomes speculative to think that the modulation of EPs by PDSs makes pathogenic bacteria sensitive towards antibiotics.

### 5.4. Biofilms Inhibition

Biofilms are an assembly of surface-integrated microbial populations enclosing an exopolysaccharide matrix [149]. Biofilm formation is a worrisome situation both in hospital environments and the food industry [150,151,152,153,154]. Comprehensive research investigating alternative plans to stop microbial biofilm formation has emphasized on the effectiveness of PDSs in limiting biofilm formation by important pathogens like *Listeria monocytogenes* [155], *Staphylococcus aureus* [156,157,158,159,160], *Pseudomonas aeruginosa* [161,162], *Escherichia coli* [163,164] and *Klebsiella pneumoniae.* PDSs, at sub-inhibitory concentrations, cause changes in the transcription of genes critical for biofilm formation, thereby contributing to antibiofilm activity [165,166,167,168,169,170]. In a study, Brackman and coworkers (2008) reported inhibitory effects of *trans*-cinnamaldehyde on biofilm formation among *Vibrio* spp. [171]. Although *trans*-cinnamaldehyde is competent enough to diminish autoinducer-2-dependent quorum sensing and biofilm production through the transcriptional regulation of specific genes, comparable transcription modulatory effects without disturbing bacterial growth have been seen in *Salmonella* [172] and *Pseudomonas aeruginosa* [173]. *Trans*-cinnamaldehyde also controls biofilm development and halts completely grown biofilms of *Cronobacter sakazakii*, found on stainless steel surfaces and feeding bottles and uropathogenic *Escherichia coli* present in urinary catheters [165,174]. Equally, terpenes like carvacrol, geraniol, thymol and EOs, exhibit potent antibiofilm activity against diverse bacterial and fungal biofilms [175,176,177]. As quorum sensing regulates the expression of genes associated with the production of virulence factors [178,179,180], focus has been given to PDSs that interfere with cell-to-cell communication, as anti-quorum-sensing molecules, which cause a decrease in the expression of virulence genes [181,182,183]. For example, the expression of *luxR* (a transcriptional regulator of quorum sensing) in *C. sakazakii* shows a decreasing trend in response to application of *trans*-cinnamaldehyde [165]. Additionally, Bodini and coworkers (2009) reported the antiquorum sensing properties of *p*-coumaric acid and garlic extracts [184]. Moreover, goldenseal, a PDS from *Hydrastis canadensis* L., disrupts microbial quorum sensing activity by interfering with the AgrCA two-component system in MRSA [185]. *Streptococcus pneumoniae* forms orderly biofilms that show persistence in human nasopharynx, permitting the development of diseases, such as pneumonia, otitis media, bacteremia and meningitis. Treating *Streptococcus pneumoniae* with 220D-F2, a plant extract from *Rubus ulmifolius*, in combination with ellagic acid and its derivatives showed a dose-dependent inhibition of biofilm formation [186]. Studies on bacterial viability following treatment with 100–200 mg/mL of 220D-F2 showed bactericidal effects as early as 3 h post inoculation for *Streptococcus pneumoniae*. Minimum inhibitory concentration (MIC) studies revealed that 80 mg/mL of 220D-F2 eliminates antibiotic resistant pneumococci strains. Testing essential oils from different plant sources, such as *Origanum vulgare*, *Mentha piperita* and *Hofmeisteria schaffneri*, against *S. aureus* has shown significant inhibitory activity. Studies on the antibiofilm activity of EOs and solvent extracts of different plants, such as *Mentha piperita*, *Pimpinella anisum* and *Coriandrum sativum*, revealed interference with the attachment of bacteria (*Staphylococcus aureus*, *Escherichia coli* and others) to the host tissues [187]. A potent antibiofilm activity against *Staphylococcus aureus* and *Escherichia coli* was observed for coriander oil at concentrations of 0.8 and 1.6 mg/mL. Non-accountable for the rise in antimicrobial resistance, carvacrol exerts an inhibitory effect, through interference with biofilm formation, against *Staphylococcus aureus* and *Staphylococcus epidermidis* [156,167]. Essential oils from *Citrus sinensis*, *Citrus bergamia*, *Sideritis erythrantha* and *Eucalyptus globulus* were found to be effective in combating vancomycin-resistant enterococci (VRE) [188,189,190,191]. In addition, a study on the antibiofilm activity of the methanolic extracts of *Sclero caryabirrea* showed that, besides inhibiting the swarming capability of *Pseudomonas aeruginosa*, they disturb quorum-sensing-mediated biofilm formation. Moreover, the extracts also exerted regulatory effects on the secretion of pyoverdine and proteases (quorum sensing reliant pathogenic factors). Growth and biofilm inhibitory effects on *Staphylococcus aureus* have also been observed for extracts obtained from *Quercus cerris* [192]. *Staphylococcus aureus* pathogenicity ranges from localized skin infection to systemic infection of different tissues. Finally, an antibiofilm activity of flavonoids from *Vaccinium macrocarpon*(cranberry) was identified against *Enterococcus faecalis*, *Escherichia coli* and *Pseudomonas aeruginosa* [193].

### 5.5. Attenuating Bacterial Virulence

The production of virulence determinants, like capsule polysaccharides, plays an important role in the development of virulence [49] in bacteria like *S. pneumoniae* [16,194,195] *Staphylococcus aureus* [196], *Klebsiella pneumoniae* and *Bacillus anthracis*. The capsule protects bacteria from phagocytosis [194]; thus, improving bacterial growth within the host [196]. Apart from virulence, the existence of a capsule helps in biofilm production and adhesion [197,198]. Capsule formation can lead to pathology even in plants; capsular polysaccharides of *Pseudomonas solanacearum*, for example, occlude xylem vessels of plants eventually causing the plant’s death. Foodborne pathogens adhere to the intestinal epithelium and invade through a receptor-mediated mechanism involving the surface proteins InlA and InlB; the presence of PDSs was found to decrease virulence factor production in *Listeria monocytogenes* [169], uropathogenic *Escherichia coli* [165] and *Salmonella enterica* serovar Enteritidis [199], reducing their invasion capacity. Additionally, capsular polysaccharides, necessary for pathogen survival inside the host, showed a reduction following exposure to PDSs [200]. Furthermore, various derivatives of salicylic acid, which is a signal molecule involved in plant protection [201], including sodium salicylate, bismuth subsalicylate and bismuth dimercaprol [202], were shown to regulate the construction of bacterial capsules. Further studies on these derivatives revealed that salicylic acid is efficient in reducing capsule manufacturing by regulating the expression of bacterial regulators of the capsule synthesis in *Staphylococcus aureus*. As quorum sensing, adhesion and capsular polysaccharides play a vital role for interaction among microbes and flourishment within the host, it becomes imperative to exploit them for therapeutics for overcoming the burden of increasing antibiotic resistance among microbes.

## 6. Hurdles to Overcome

Regardless of their antimicrobial effects, the use of PDSs as alternatives or complements (either for increasing the activity or decreasing the dose) to antibiotics is impeded by regulatory factors that pose a challenge regarding their implementation in the treatment of diseases. One challenge is the development of effective extraction and purification systems for the isolation of newer and safer plant-derived antimicrobials [203]. In addition to solvent extractions systems, microwave and ultrasonic-assisted extraction methods are currently being explored for the extraction of PDSs [204]. Screening bioactive compounds for elucidating their structure, function and action mechanisms is necessary to fine tune their production and maximize their performance with simplicity, specificity and speed. Isolation and purification processes have been accelerated with the development of high-pressure liquid chromatography (HPLC) and a variety of spectroscopic techniques (UV-visible, infrared (IR), nuclear magnetic resonance (NMR) and mass spectroscopy [205]. Additionally, reduction in the production cost can be achieved by rationalizing their chemical synthesis and further increasing their efficacy by modifying their structures. To properly address the feasibility of the use of PSDs, the following points need to be considered: (1) dose in terms of bacteriostatic/-cidal effect; (2) variation in phytochemical constituents; (3) safety in humans; and (4) efficacy without any concurrent effect in terms of resistance development. A deeper insight into the toxicity of PDSs, their mechanism of action and influential interactions with antibiotics needs further exploration to have a complete understanding of their role in disease treatment and for their broader adoption in the near future. A recent strategic approach, using fluorescently labeled antibiotics, promises good results in resistance and toxicological studies and in elucidating the potency of PDSs in the treatment of different diseases [206]. Though much attention has been paid to adding flexibility to approval strategies, consistency in safety, efficacy and delivery methods is required for their commercialized use against broad range of pathogens.

## 7. Conclusions

The phenomenon of antibiotic resistance is not only old but is also a very difficult situation that emerges among bacteria exposed to antibiotics. Sadly, it has not been given a priority even though it has become a serious issue concerning human health. This worrisome situation is continuously growing, but still, it has not been included in planning strategies among health experts, the scientific community or researchers. The current crisis of rising multidrug resistance in bacteria is creating a global threat, necessitating the development of new alternatives. With the relative absence of new antimicrobials in the market, the sleeping giants of the pharmaceutical industry (medicinal herbs) have high potential for being a source of natural drugs that can be utilized to combat the menace created by antibiotic resistance. Plant-based antimicrobials have a potential for use in the manufacturing of drugs. Exhibiting a potent antimicrobial activity, plant-based antimicrobials, either alone or combined with antibiotics, can help in dealing with the present crisis of antibiotic resistance. A study by Carradori et al. shows antimicrobial activity of *Crocus sativus-derived* compounds against different bacterial sp. [207]. This study shows the promising antimicrobial activity from the modified compounds of *Crocus sativus.* The MIC values obtained in a particular activity were showing synergy among *Crocus sativus* components. These kinds of studies provide an insight that certain chemical and structural modifications can provide us with more promising and effective compounds that can help in the formulation of innovative drugs to fight against antifungal and antimicrobial situation. In conclusion, there is an urgent requirement to develop plant-based antimicrobials to counteract antibiotic resistance. The search for natural compounds, extracted from medicinal plants, is a necessity. Research based on ethnopharmacology the field of study that uses plant herbs as a therapeutic option, may be used as a guide in our search for suitable agents against the spread of antibiotic resistance. The global nature of this crisis and its substantial health and economic burdens prompt us to urgently identify new alternatives as well as implement new policies to combat antibiotic resistance.

## Figures and Tables

**Table 1:** Plant-derived substances and their action against different microbes.

Plant Name	Plant Derivatives	Bacterial sp.	* MIC Value	References
*Anogeissusa cuminata*	terpenoids, flavonoids, saponins, tannins, alkaloids	*S. aureus*	0.29 mg/mL	[83]
		*A. baumannii*	1.51 mg/mL	
		*C. freundii*	3.41 mg/mL	
		*E. coli*	3.41 mg/mL	
		*K. oxytoca*	1.51 mg/mL	
		*K. pneumoniae*	0.67 mg/mL	
		*P. aeruginosa*	0.67 mg/mL	
*Azadirachta indica*	β-sitosterol, flavonoids	*S. aureus*	3.41 mg/mL	[83] [84]
		*A. baumannii*	4.27 mg/mL	
		*C. freundii*	3.41 mg/mL	
		*E. coli*	4.27 mg/mL	
		*K. oxytoca*	9.63 mg/mL	
		*K. pneumoniae*	4.27 mg/mL	
		*P. aeruginosa* *H. pylori*	9.63 mg/mL 128 µg/mL	
*Bauhinia variegata*	terpenoids, flavonoids, tannins, saponins, glucoside,	*S. aureus*	3.41 mg/mL	[83]
		*A. baumannii*	9.63 mg/mL	
		*C. freundii*	9.63 mg/mL	
		*E. coli*	4.27 mg/mL	
		*K. oxytoca*	9.63 mg/mL	
		*K. pneumoniae*	4.27 mg/mL	
		*P. aeruginosa*	3.41 mg/mL	
*Boerhaavia diffusa*	β-sitosterol, flavonoids	*S. aureus*	4.27 mg/mL	[83]
		*A. baumannii*	9.63 mg/mL	
		*C. freundii*	9.63 mg/mL	
		*E. coli*	NA	
		*K. oxytoca*	9.63 mg/mL	
		*K. pneumoniae*	9.63 mg/mL	
		*P. aeruginosa*	9.63 mg/mL	
*Punica granatum*	flavonoids, ellagitannin, punicalagin, ellagic acid	*S. aureus*	0.29 mg/mL	[83]
		*A. baumannii*	3.41 mg/mL	
		*C. freundii*	0.67 mg/mL	
		*E. coli*	0.67 mg/mL	
		*K. oxytoca*	3.41 mg/mL	
		*K. pneumoniae*	3.41 mg/mL	
		*P. aeruginosa*	0.67 mg/mL	
*Soymida febrifuga*	methyl angolensate, luteolin 7-O-glucoside, flavonoid, sitosterol, myricetin	*S. aureus*	067 mg/mL	[83]
		*A. baumannii*	1.51 mg/mL	
		*C. freundii*	3.41 mg/mL	
		*E. coli*	4.27 mg/mL	
		*K. oxytoca*	3.41 mg/mL	
		*K. pneumoniae*	3.41 mg/mL	
		*P. aeruginosa*	4.27 mg/mL	
*Terminalia chebula*	flavonoids and flavins, terpenoids, steroids, alkaloids, tannins and their derivatives, glycosides	*S. aureus*	1.51 mg/mL	[83]
		*A. baumannii*	9.63 mg/mL	
		*C. freundii*	NA	
		*E. coli*	9.63 mg/mL	
		*K. oxytoca*	9.63 mg/mL	
		*K. pneumoniae*	9.63 mg/mL	
		*P. aeruginosa*	9.63 mg/mL	
*Tinospora cordifolia*	alkaloids terpenoids, lactones, glycosides, steroids, phenolics	*S. aureus*	4.27 mg/mL	[83]
		*A. baumannii*	NA	
		*C. freundii*	9.63 mg/mL	
		*E. coli*	4.27 mg/mL	
		*K. oxytoca*	9.63 mg/mL	
		*K. pneumoniae*	9.63 mg/mL	
		*P. aeruginosa*	4.27 mg/mL	
*Tribulus terrestris*	flavonoids, flavonol glycosides, steroidal saponins and alkaloids	*S. aureus*	3.41 mg/mL	[83]
		*A. baumannii*	9.63 mg/mL	
		*C. freundii*	9.63 mg/mL	
		*E. coli*	4.27 mg/mL	
		*K. oxytoca*	4.27 mg/mL	
		*K. pneumoniae*	9.63 mg/mL	
		*P. aeruginosa*	3.41 mg/mL	
*Petroselinum crispum EO (Essential oil)*	phenolic, flavonoids, coumarins	*B. cereus*	22.68 µL/mL	[85]
		*S. aureus*	10.80 µL/mL	
		*P. aeruginosa*	47.62 µL/mL	
		*E. coli*	10.80 µL/mL	
		*S. typhimurium*	47.62 µL/mL	
*Levisticum officinale EO*	terpenoids, n-butylidene phthalide n-butyl-phthalide, sedanonic anhydride, d-terpineol, l, l phenolic,	*B. cereus*	47.62 µL/mL	[85]
		*S. aureus*	2.45 µL/mL	
		*P. aeruginosa*	22.68 µL/mL	
		*E. coli*	10.80 µL/mL	
		*S. typhimurium*	47.62 µL/mL	
*Occimomum basilicum EO*	rosmarinic acid, phenol and terpenoid	*B. cereus*	10.80 µL/mL	[85]
		*S. aureus*	2.45 µL/mL	
		*P. aeruginosa*	22.68 µL/mL	
		*E. coli*	10.80 µL/mL	
		*S. typhimurium*	22.68 µL/mL	
*Thymus vulgare EO*	p-cymene, γ-terpinene, thymol	*B. cereus*	0.56 µL/mL	[85]
		*S. aureus*	0.06 µL/mL	
		*P. aeruginosa*	0.56 µL/mL	
		*E. coli*	0.27 µL/mL	
		*S. typhimurium*	0.56 µL/mL	
*Cannabis sativa* L., *EO*	*phenol*, *flavonoid*	*S. aureus*	8 mg/mL	[86]
*Acrosta phylosuvaursi*	ellagic and gallic acid tannins, flavonoids, phenol	*S. aureus*	90 µg/mL	[87]
*Coptis chinensis*	isoquinoline, alkaloids		121 µg/mL	
*Eucalyptus globulus*	1,8-cineole, α-pinene, p-cymene		118 µg/mL	
*Larreatri dentata*	alkaloids, lignans, flavonoid, terpenoids		60 µg/mL	
*Alpinia galanga*	α-pinene, myrcene, limonene	*Mycobacterium smegmatis* mc2 155	3.12–25 mg/L	[88]
*Ammannia* spp.	dioxyflavanol, quercetin and kaempferol	*E. coli*	125 µg/mL	[89]
*Berberis vulgaris*	isoquinoline, alkaloids,	*P. aeruginosa*	250–1000 µg/mL	[90]
*Acer saccharum*	flavonoids, tannins	*E. coli*	5 and 10 mg/mL	[91]
		*P. aeruginosa*		
		*P. mirabilis*		
*Catharanthus roseus*	limonene, terpenoid	*P. aeruginosa*	25 mg/L	[92]
*Holarrhena antidysenterica*	triterpenoids, sitosterol, phytosterol	*P. aeruginosa*	20 mg/L	[93]
*Cuminum cyminum*	alkaloid, phenols, flavonoid, glycoside, saponin, tannin and steroid	*S. aureus*	5 mg/mL	[94]
*Salvia fruticosa*	flavonoids, phenolics and rosemarinic acid	*S. epidermidis*	5 µl/mL	[95]
*Chenopodium Ambrosioides*	α-terpinene, ascaridole	*S. aureus*	170.6 µl/mL	[96]
*Terminalia chebola*	ellagic acid, gallic acid	*E. coli*	12.1–97.5 µg/mL	[97]
*Persea lingue*	flavonoids,	*S. aureus*	1.56 mg/L	[98]
*Ipomoea muricata*	ipomine, ipalbine, ipalbidine and ipalbinium	*E. coli*	10 µg/mL	[99]
*Hypericum olympicum*	essential oils, α-pinene, β-ocimene, β-caryophyllene, germacrene-D	*S. aureus*	50 µM	[100]
*Alkanna orientalis*	β-eudesmol, α-eudesmol and γ-eudesmol	*S. aureus*	100 µM	[101]
*Eucalyptus tereticornis*	saponins, tannins, steroids flavonoids, cardiac glycosides	*E. coli*	25 and 50 µg/mL	[89]
*Salvia officinalis*	phenols, terpenoids	*E. cloacae*, *E. coli*, *S. typhimurium*, *P. aeruginosa*, *B. cereus*	0.01 mg/mL, 0.045 mg/mL, 0.045 mg/mL, 0.045 mg/mL, 0.09 mg/mL	[102]

* MIC corresponds to effect observed for the plant extract against given bacterial isolates.

**Table 2:** Mechanism of action of plant-derived substances (PDSs) against broad range of microbes.

Phytochemicals	Extract	Mode of Action	Antimicrobial Resistant Microbes	References
Flavonoids	*Vaccinium macrocarpon* Alt (cranberry)	Modifies biofilm formation	*Enterococcus faecalis*, *E. coli*, *Pseudomonas aeruginosa*	[114,115,116]
	Myricetin, robinetin, epigallocatechin	Blocks bacterial DNA synthesis	*E. coli*	[117]
	Quercetin	Inhibit ATPase activity, GrYB protein, elevates extracellular phosphatase and β-galactosidase	*E. coli*, *S. aureus*	[118]
Plant-derived peptides	*Moringa oleifera*	Membrane disruption	*E. coli*, *S. aureus*, *P. aeruginosa*, *S. Typhimurium*	[119]
Essential oils (EOs)	*Petroselinum crispum* EO, *Levisticum officinale* EO, *Ocimum basilicum* EO, *Thymus vulgaris* EO *Cannabis sativa* L., EO	Increase cell permeability, leakage of cell constituents, alteration of bacterial cell wall and membrane disturbance, ATP loss, inhibit protein synthesis, lead to pH disturbance, intracytoplasmic damage, DNA damage, inhibit quorum sensing Inhibits biofilm formation	*Bacillus cereus*, *Staphylococcus aureus*, *P. aeruginosa*, *E. coli*, *S. Typhimurium* *S. aureus*	[120,121] [102]
Tea tree oil (TTO)	terpenes, monoterpenes, sesquiterpenes	disrupts membrane permeability, damages cell membrane, obstructs cell growth, cause cell death	*E. coli*, *S. aureus*, *C. albicans*	[122,123]
Natural Efflux pump inhibitors (EPIs)	reserpine, gallotannin, piperine, curcumin, berberine, chalcones, carnosic acid	Inhibit various efflux pump in bacteria (EtBr EP, MexAB-OprM)	MDR Uropathogenic *E. coli*, MDR, *P. aeruginosa* (clinical isolates)	[82,90,97,121,124,125]
